# Cancer screening: the collateral damage of the pandemic in Egypt

**DOI:** 10.1186/s42506-021-00073-2

**Published:** 2021-06-02

**Authors:** Abdullah S. Eldaly

**Affiliations:** Department of Plastic and Reconstructive Surgery, Tanta University Hospitals, Tanta, Egypt

To the Editor,

Severe acute respiratory syndrome coronavirus 2 (SARS-CoV-2) pandemic has cast a shadow on all cancer screening programs; millions of screening appointments were canceled due to the pandemic and resulting in depriving the population from a vital healthcare service that could save lives. One study in the USA reported a drastic drop in the screenings for breast, colon, and cervical cancer by 94%, 86%, and 94% respectively between January 2020 and April 2020 [1]. In Japan, hepatocellular carcinoma (HCC) kept scheduled visits dropped dramatically from 97% before February 2020 to 49.1% in May 2020 according to one study [2]. The consequences of these canceled or postponed appointments will soon come to the surface and healthcare workers should be prepared for. In Egypt, the relatively weak screening programs for liver and breast cancer are expected to be the most severed as a direct result of understaffing, the fear of visiting the screening clinics, as well as turning many general hospitals and cancer institutes into SARS-CoV-2 isolation centers.

## HCC screening

Hepatocellular carcinoma (HCC) is the fourth most common cause of cancer-related mortality in the world [1]. The most common cause of HCC is liver cirrhosis of any etiology.

Egypt has the second highest incidence rate of HCC in the world with an age-standardized rate (ASR) of 32.2 [3]. This heavy burden is a direct result of high HCV prevalence among Egyptians and is due to the government’s parenteral anti-schistosomal therapy (PAT) mass-treatment campaigns that took place between the 1950s and 1980s and lead to transmission of HCV throughout Egypt [4].

HCC is clinically silent in most patients until late stages and diagnosis could be challenging. Patients with tumors of a size of 2 cm or less could theoretically benefit from all treatment options while those with larger tumors or extra-hepatic spread have limited options and suffer worse prognosis. Screening for HCC in high-risk individuals was started in the 1980s in order to early detect the tumor and possibly improve the outcomes [5]. The most recent screening guidelines recommend that all individuals at risk of HCC should undergo an alpha fetoprotein (AFP) test as well as abdominal ultrasonography at regular 6-month intervals.

The benefits of HCC surveillance programs were well established in 2004 when a randomized clinical trial conducted in China revealed that biannual screening using AFP and ultrasonography had reduced HCC mortality by 37% [6]. Moreover, the emergence of new effective treatments for early HCC in the last two decades has most likely increased the benefits from surveillance programs. HCC surveillance is now considered a cornerstone of the optimum care to cirrhotic patients and the vast majority of physicians would recommend screening for high-risk patients.

Although Egypt has a heavy burden of liver cirrhosis and HCC, most of the government's attention is directed to HCV screening and eradication; a great success was achieved during the national campaign of HCV screening and eradication in 2018 and the country is moving steadily toward the elimination target [7]. However, cirrhotic patients remain at risk of developing HCC even after HCV eradication which means millions of Egyptians are still at risk of developing HCC despite the great success of the HCV eradication campaigns.

In Egypt, the weak HCC surveillance system is expected to be severed due to the pandemic. Over 300 public hospitals were turned into isolation centers for SARS-CoV-2 patients including many tropical medicine hospitals and some cancer and liver institutes [8]. The exact influence of the pandemic on the HCC surveillance program in Egypt is not exactly known because we lack enough data. However, it is not expected to differ significantly from the worldwide trend which means we are risking thousands of early HCC cases to pass unnoticed or be discovered when it is too late.

## Breast cancer screening

With a staggering 200–400% increase in the relative incidence of breast cancer in Egypt between the years 1990 and 2017—an increase that was only surpassed by the Kingdom of Saudi Arabia—the deadly cancer has declared itself a challenging public health problem that needs urgent intervention [9]. This is, unfortunately, a part of the bigger picture where the burden of both cases and deaths is shifting steadily toward the less developed world as a direct result of urbanization and the resulting obesity and diabetes [10]. The survival depends greatly on the country’s economic status: a study comparing cancer survival in five continents reported varying 5-year survival rates of 80%, 60%, and 40% in high-income, middle-income, and low-income countries, respectively [11].

In Africa, the continent with the highest breast cancer mortality rates, the situation is becoming increasingly challenging [12]. The rapid industrialization without an equivalent growth in healthcare services and infrastructure has left African women with breast cancer more vulnerable than their peers in any other part in the world. The lack of screening, education, and campaigning lead to a great difference in staging at time of presentation between African and European women: about 50–70% of African women with breast cancer present with grade III or IV, while European women tend to present at stage I and II [13–15]. Ductal carcinoma is the most common histological type in Africa as well as the rest of the world. However, medullary and mucinous breast carcinomas are more reported in African women; these histological types tend to have poorer prognosis than ductal carcinoma. Moreover, the rates of the triple negative breast cancer (TNBC), which has the worst prognosis among other molecular subtypes, are significantly higher in African women [16]; this for sure adds to the aforementioned problem of late stages at presentation. Women with advanced stages are less likely to benefit from a breast conserving surgery leaving them to mastectomy, adjuvant hormonal therapy, chemotherapy, or even palliation. Women with TNBC are unfortunately less likely to respond to adjuvant hormonal therapy and chemotherapy which along with the late stages at presentation contributes to the suffering of African women with breast cancer [17].

Breast cancer is the most expensive cancer to treat [18]; this economic burden has important consequences on both the patients and the weak healthcare systems in the developing world. Breast cancer survivors, especially those who received adjuvant hormonal therapy suffer worsening financial status even with receiving financial assistance [19, 20]. However, this financial burden could be greatly lowered through early detection since the cost of treatment is inversely related to the stage at presentation. A return-on-investment (ROI) analysis of a breast cancer screening program in Egypt found that the average per-person treatment cost for screened and unscreened patients was estimated to be $28,632 and $58,170, respectively, with a cumulative lifetime risk of 6.36%. Total screening program cost per person was $112.10. The study estimated an expected decrease in late-stage breast cancer diagnosis by 13.7% as a result of the screening program, saving $4049 in treatment costs per individual diagnosed. The analysis resulted in a positive ROI of 133% for facility-based screening [21]. This is the economic rationale of the mass screening campaign for breast cancer that is currently running in Egypt.

Despite the initial success of the national campaign of breast cancer screening, the campaign was severed as the pandemic took over. Many hospitals that were participating in the campaign were turned into isolation centers for SARS-CoV-2 patients. Moreover, the understaffing that happened in other non-isolation hospitals affected the service in the outpatient clinics where women are supposed to be checked annually for breast cancer. Another important factor that markedly influenced breast cancer screening during the pandemic is the fear of getting infected with SARS-CoV-2 while doing the annual breast check in the hospital.

## What is next?

There is no doubt Egypt needs to adopt new efficient strategies for cancer screening, but for now, we should aim to restore our pandemic-exhausted system first. The first step toward restoring the normal capacity of the healthcare system is to speed up the vaccination process. Unfortunately, Egypt has been performing poorly regarding the SARS-CoV-2 vaccination with only 164,534 doses administrated by April 2021 [22]. This weak performance will not only lead to more patients with SARS-CoV-2 exhausting the healthcare system but also will affect almost every chronic/high-risk patient including those who undergo regular cancer screening.

## Data Availability

Not applicable.

## Abbreviations

SARS-CoV-2: Severe acute respiratory syndrome coronavirus 2
HCC: Hepatocellular carcinoma
HCV: Hepatitis C virus
HBV: Hepatitis B virus
DNA: Deoxyribonucleic acid
ASR: Age-standardized rate
PAT: Parenteral anti-schistosomal therapy
AFP: Alpha fetoprotein
TNBC: Triple negative breast cancer
ROI: Return-on-investment
